# Supplementary material for: A Natural CHI3L1—Targeting Compound, Ebractenoid F, Inhibits Lung Cancer Cell Growth and Migration and Induces Apoptosis by Blocking CHI3L1/AKT Signals
Source: Molecules. 2022 Dec 31;28(1):329. doi: 10.3390/molecules28010329 (PMC9822003; doi:10.3390/molecules28010329)
Supplement: Supplementary file 1 [file molecules-28-00329-s001.zip › molecules-2048632-supplementary.pdf]

Supporting information

A Natural CHI3L1−-Targeting Compound, Ebractenoid F, Inhibits Lung Cancer Cell Growth and Migration and Induces Apoptosis by Blocking CHI3L1/AKT Signals

Da Eun Hong [1], Ji Eun Yu [1], Jin Woo Lee [1], Dong Ju Son [1], Hee Pom Lee [1], Yuri Kim [1], Ju Young Chang [1], Dong Won Lee [1], Won Kyu Lee [2], Jaesuk Yun [1], Sang Bae Han [1], Bang Yeon Hwang [1,*] and Jin Tae Hong [1,*]

[1] College of Pharmacy & Medical Research Center, Chungbuk National University, Osongsaengmyeong 1-ro 194-21, Osong-eup, Heungduk-gu, Cheongju, Chungbuk, 28160, Republic of Korea
[2] Department of New Drug Development Center, Osong Medical Innovation Foundation (KBio Health), Cheongju, South Korea
* Correspondence: jinthong@chungbuk.ac.kr; Tel.: +82-43-261-2813; Fax: +82-43-268-2732,

Supplementary Figure S1.

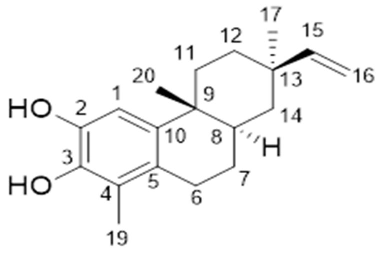

Ebractenoid F
Chemical Formula : $C_{19}H_{26}O_2$
Exact Mass : 286.19

Figure S1. Structure of ebractenoid F.

Regarding the identification of the chemical structure, the device analysis data were organized and prepared.

Ebractenoid F: Light yellow powder; ESIMS *m/z* 285 $[M-H]^-$; ^{1}H-NMR (500 MHz, $CDCl_3$) δ_H 6.73 (1H, s, H-1), 5.89 (1H, dd, *J* = 17.5, 11.0 Hz, H-15), 5.00 (1H, dd, *J* = 17.5, 1.0 Hz, H-16a), 4.92 (1H, dd, *J* = 11.0, 1.0 Hz, H-16b), 2.68 (2H, m, H-6), 2.14 (3H, s, H-19), 1.97 (1H, m, H-11a), 1.77 (1H, m, H-11b), 1.80-1.60 (2H, m, H-7), 1.65 (1H, m, H-8), 1.55-1.41 (2H, m, H-12), 1.47 (1H, t, *J* = 13.0 Hz, H-14a), 1.23 (1H, m, H-14b), 1.05 (3H, s, H-17), 1.03 (3H, s, H-20); ^{13}C-NMR (125 MHz, $CDCl_3$) δ_C 108.8 (C-1), 140.8 (C-2), 140.4 (C-3), 122.7 (C-4), 127.1 (C-5), 26.9 (C-6), 25.7 (C-7), 36.4 (C-8), 36.4 (C-9), 139.9 (C-10), 34.1 (C-11), 32.9 (C-12), 36.4 (C-13), 39.7 (C-14), 151.1 (C-15), 108.8 (C-16), 22.8 (C-17), 11.4 (C-19), 21.3 (C-20).

Supplementary Figure S2.

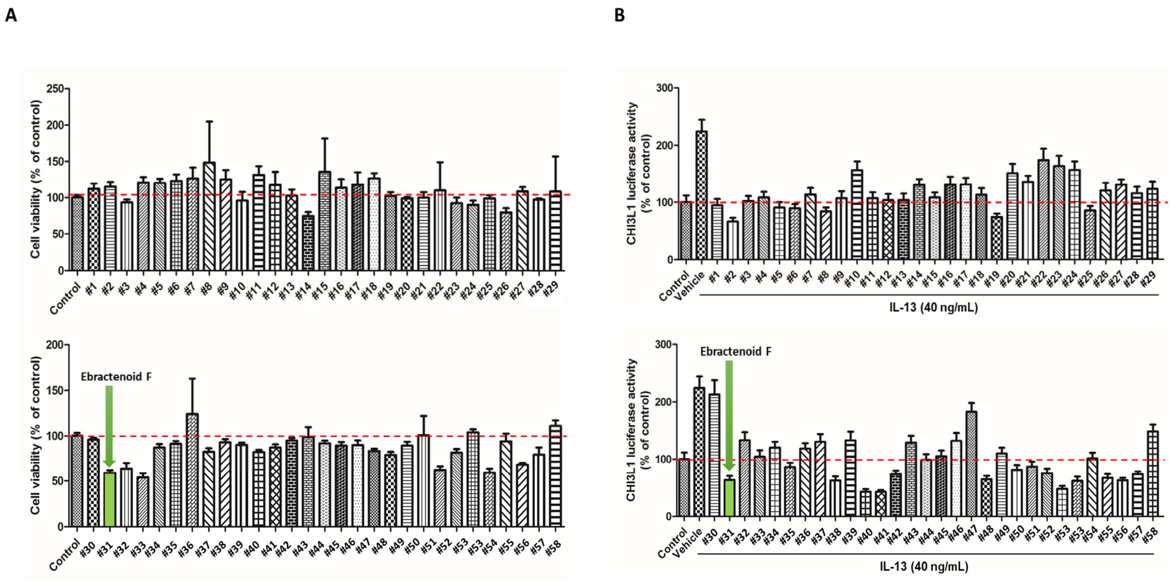

Figure S2. Ebractenoid F was selected as a useful substance targeting CHI3L1.

Among the 58 natural substance candidates, experiments were conducted to select substances that could effectively inhibit cell proliferation and transcription activity of CHI3L1 in A549 lung cancer cells. MTT assay was performed to confirm the effect of inhibiting cell proliferation of 58 substances (**A**). Luciferase assay was performed to find out the transcription activity of CHI3L1 in inhibiting cell proliferation of 58 substances. IL-13 stimulation (40 ng/mL) CHI3L luciferase analysis (**B**). All 58 substances used in the experiment were dissolved in DMSO and used at 10 μg/ml. All of the experiments were conducted more than three times.

Supplementary Figure S3.

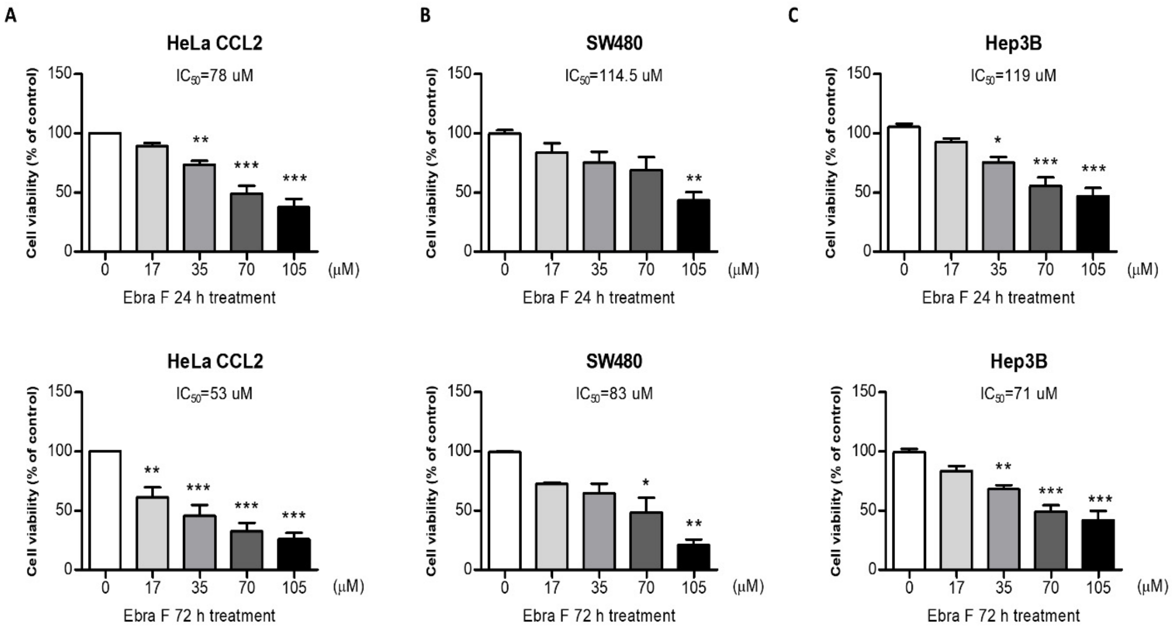

Figure S3. Effect of ebractenoid F on various cancer cell growth.

The effect of ebractenoid F on HeLa CCL2 (cervical cancer cells) (**A**), SW480 (colon cancer cells) (**B**) and Hep3B (liver cancer cells) (**C**) cells observed using the cell viability assay (MTT assay) after 24 h and 72 h. The data were expressed as the mean ± S.D. of three experiments. *, $P \leq 0.05$; **, $P<0.01$; ***, $P<0.001$ indicates statistically significant differences from the control group.

Supplementary Figure S4.

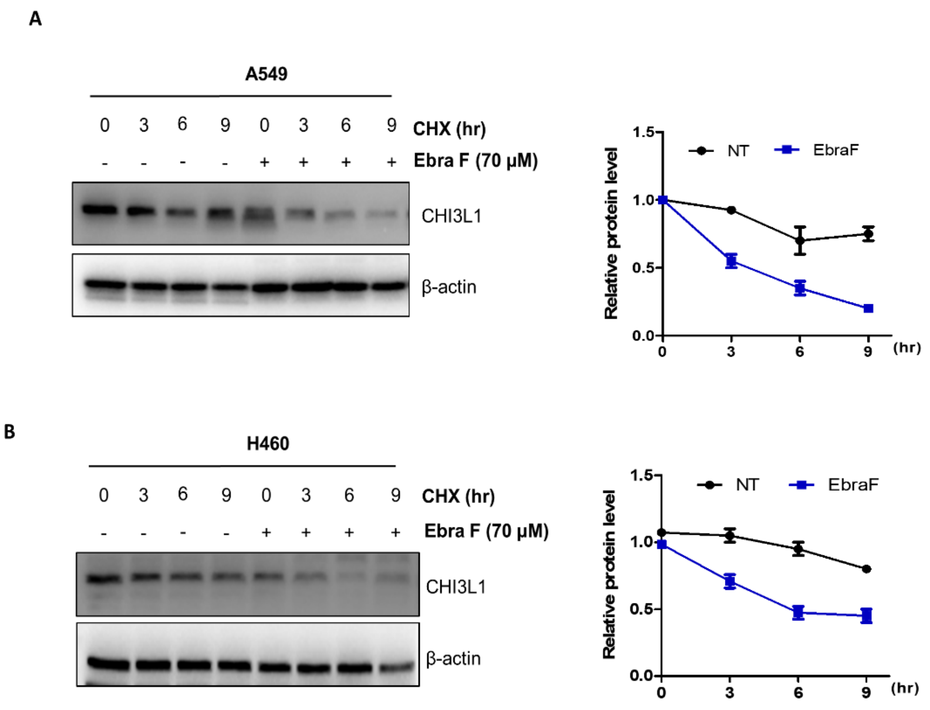

Figure S4. Analysis of protein stability by the cycloheximide chase assay and the mRNA expression of CHI3L1. Lung cancer cells were treated with protein synthesis inhibitor cycloheximide (CHX) with or without ebractenoid F for indicated lengths of time. CHI3L1 protein level was measured by Western blotting analysis. The data were expressed as the mean ± S.D. of three experiments.

Supplementary Figure S5.

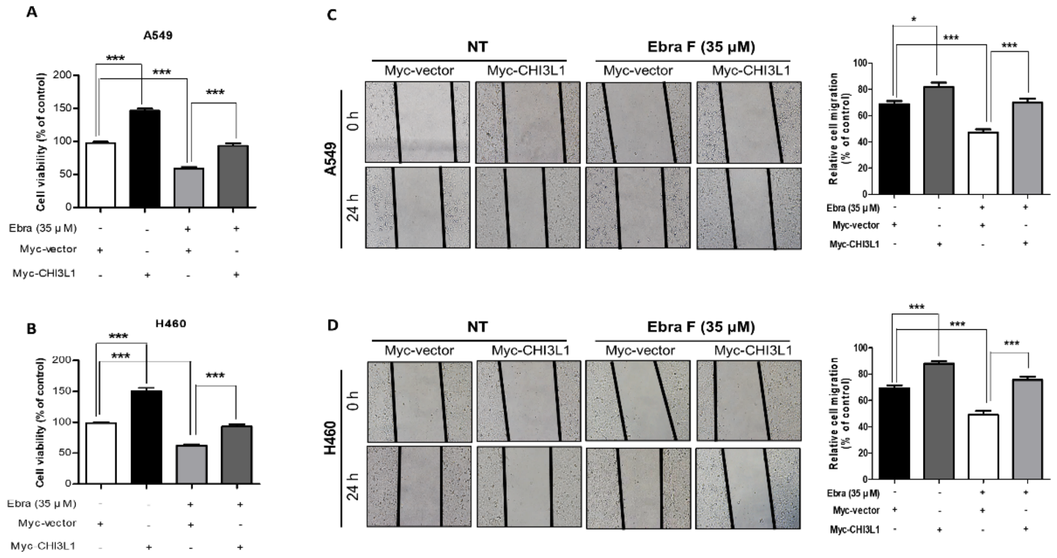

Figure S5. Proliferation and migration rescue assay. Lung cancer cells were treated with ebractenoid F and then transfected with the myc-CHI3L1 plasmid or control plasmid (myc-vector). Cell proliferation was observed using the cell viability assay (MTT assay) (**A** and **B**). Cell migration was observed using wound healing assay, which was performed in the same way as previously mentioned (**C** and **D**). The data were expressed as the mean ± S.D. of three experiments. *, P ≤ 0.05; ***, P<0.001 indicates statistically significant differences from the control group.

Supplementary Figure S6.

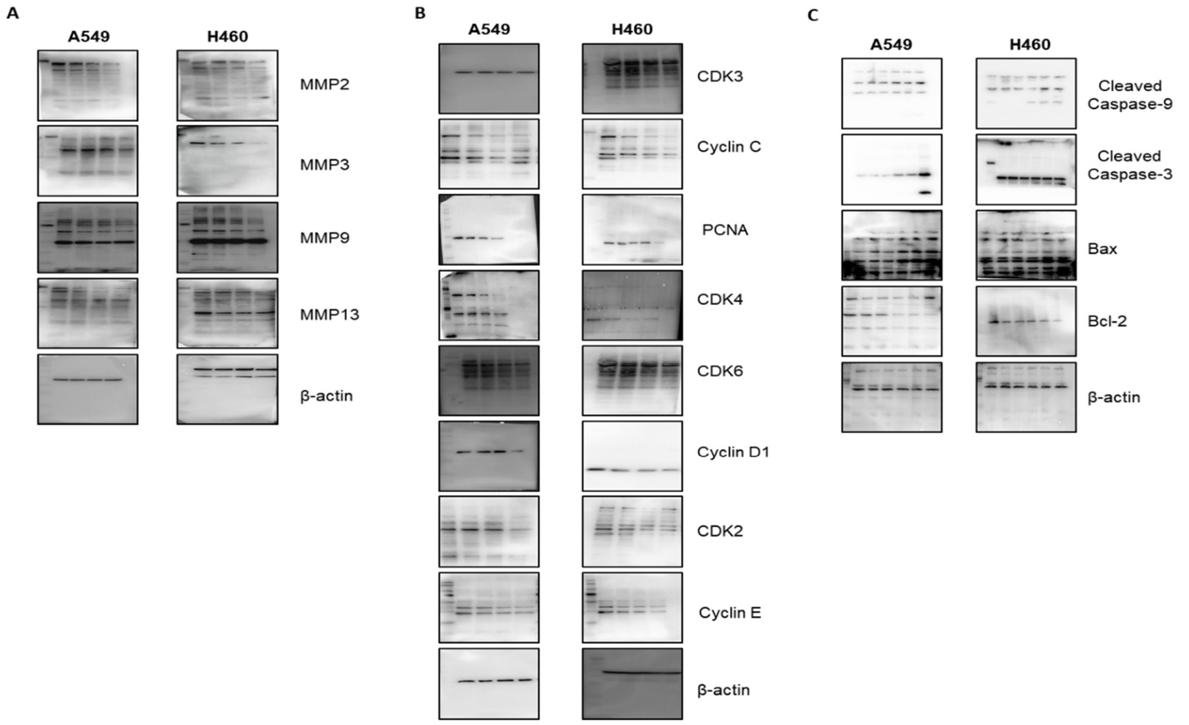

Supplementary Figure S6.

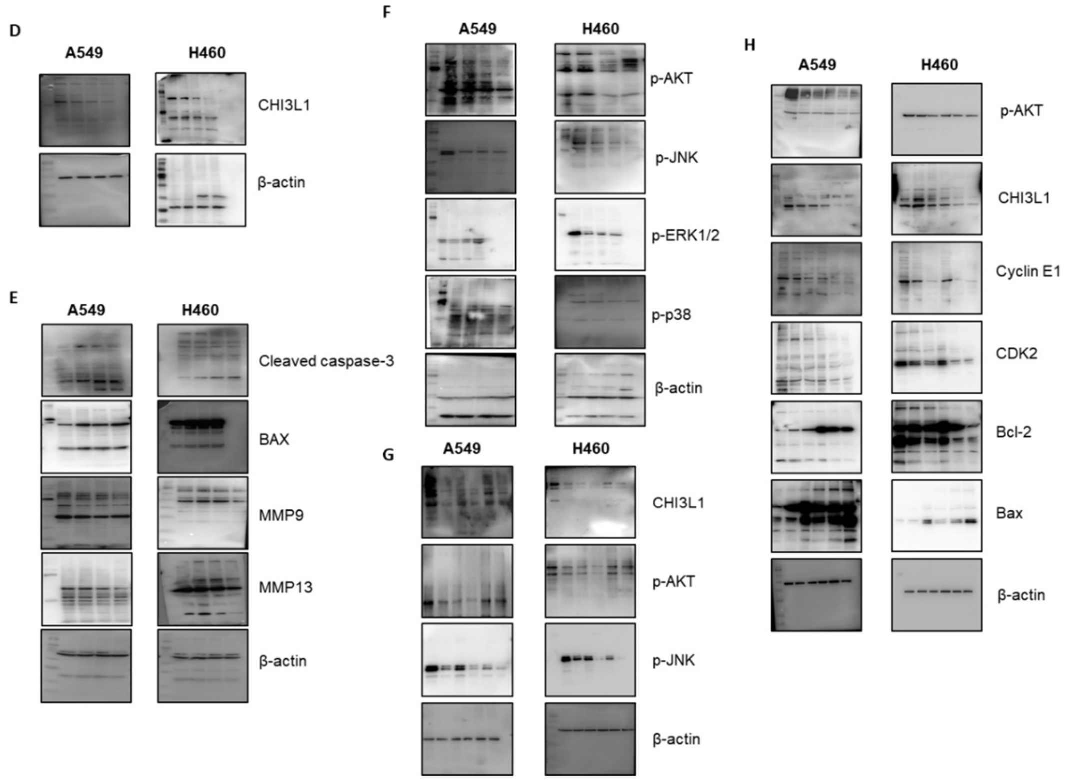

Figure S6. The whole Western blot with markers.
